# A Microfiltration Device for Urogenital Schistosomiasis Diagnostics

**DOI:** 10.1371/journal.pone.0154640

**Published:** 2016-04-28

**Authors:** Yuan Xiao, Yi Lu, Michael Hsieh, Joseph Liao, Pak Kin Wong

**Affiliations:** 1 Department of Biomedical Engineering, The Pennsylvania State University, University Park, PA, United States of America; 2 Department of Aerospace and Mechanical Engineering, The University of Arizona, Tucson, AZ, United States of America; 3 Department of Urology, Stanford University School of Medicine, Stanford University, Stanford, CA, United States of America; 4 Veterans Affairs Palo Alto Health Care System, Palo Alto, CA, United States of America; 5 Department of Mechanical and Nuclear Engineering and Department of Surgery, The Pennsylvania State University, University Park, PA, United States of America; Texas A&M University, UNITED STATES

## Abstract

Schistosomiasis is a parasitic disease affecting over 200 million people worldwide. This study reports the design and development of a microfiltration device for isolating schistosome eggs in urine for rapid diagnostics of urogenital schistosomiasis. The design of the device comprises a linear array of microfluidic traps to immobilize and separate schistosome eggs. Sequential loading of individual eggs is achieved autonomously by flow resistance, which facilitates observation and enumeration of samples with low-abundance targets. Computational fluid dynamics modeling and experimental characterization are performed to optimize the trapping performance. By optimizing the capture strategy, the trapping efficiency could be achieved at 100% with 300 μl/min and 83% with 3000 μl/min, and the filtration procedure could be finished within 10 min. The trapped eggs can be either recovered for downstream analysis or preserved *in situ* for whole-mount staining. On-chip phenotyping using confocal laser fluorescence microscopy identifies the microstructure of the trapped schistosome eggs. The device provides a novel microfluidic approach for trapping, counting and on-chip fluorescence characterization of urinal *Schistosoma haematobium* eggs for clinical and investigative application.

## Introduction

Schistosomiasis is a parasitic disease caused by schistosomes. It is one of the most devastating tropical diseases affecting over 200 million people in Africa, the Middle East, Asia, and South America [1, 2]. *Schistosoma haematobium*, which causes urogenital schistosomiasis, accounts for the majority of schistosome infections worldwide. During the dissemination, the eggs of *Schistosoma haematobium* are excreted into urine, and are characterized by the oval-shaped body and external terminal spine with a miracidium inside [3]. Urogenital schistosomiasis is generally diagnosed by microscopic inspection of schistosome eggs in urine.

Rapid characterization of schistosome eggs represents a key step for clinical management of schistosomiasis. Due to the low concentration of schistosome eggs and matrix effects of urine [4, 5], cumbersome sample preparation procedures, such as centrifugation, filtration, washing and staining, are required for the characterization of schistosome eggs [6, 7]. Diagnosis of schistosomiasis are continually being improved by other emerging diagnostic techniques, such as fluorescence staining, polymerase chain reaction, and electrochemical biosensors [8, 9]. Nevertheless, the low abundance of schistosome eggs in urine remains a major bottleneck of urogenital schistosomiasis diagnostics.

The advent of microfluidics offers new opportunities in the analysis of parasite eggs toward point-of-care applications [9–12]. For instance, a micromesh based microfluidic device was developed for high throughput collection of *Cryptosporidium parvum* eggs in water [13]. Serpentine channels with cross-flow traps were designed for studying drosophila eggs [11]. For diagnosis of urogenital schistosomiasis, a large throughput with high trapping efficiency for egg analysis and enumeration is required due to the low abundance of schistosome eggs in urine. The device should also be inert to matrix effects and operate directly with urine. Furthermore, the device should allow in situ microscopic characterization and, ideally, retrieval of the trapped eggs for downstream molecular analysis.

**Fig 1:**
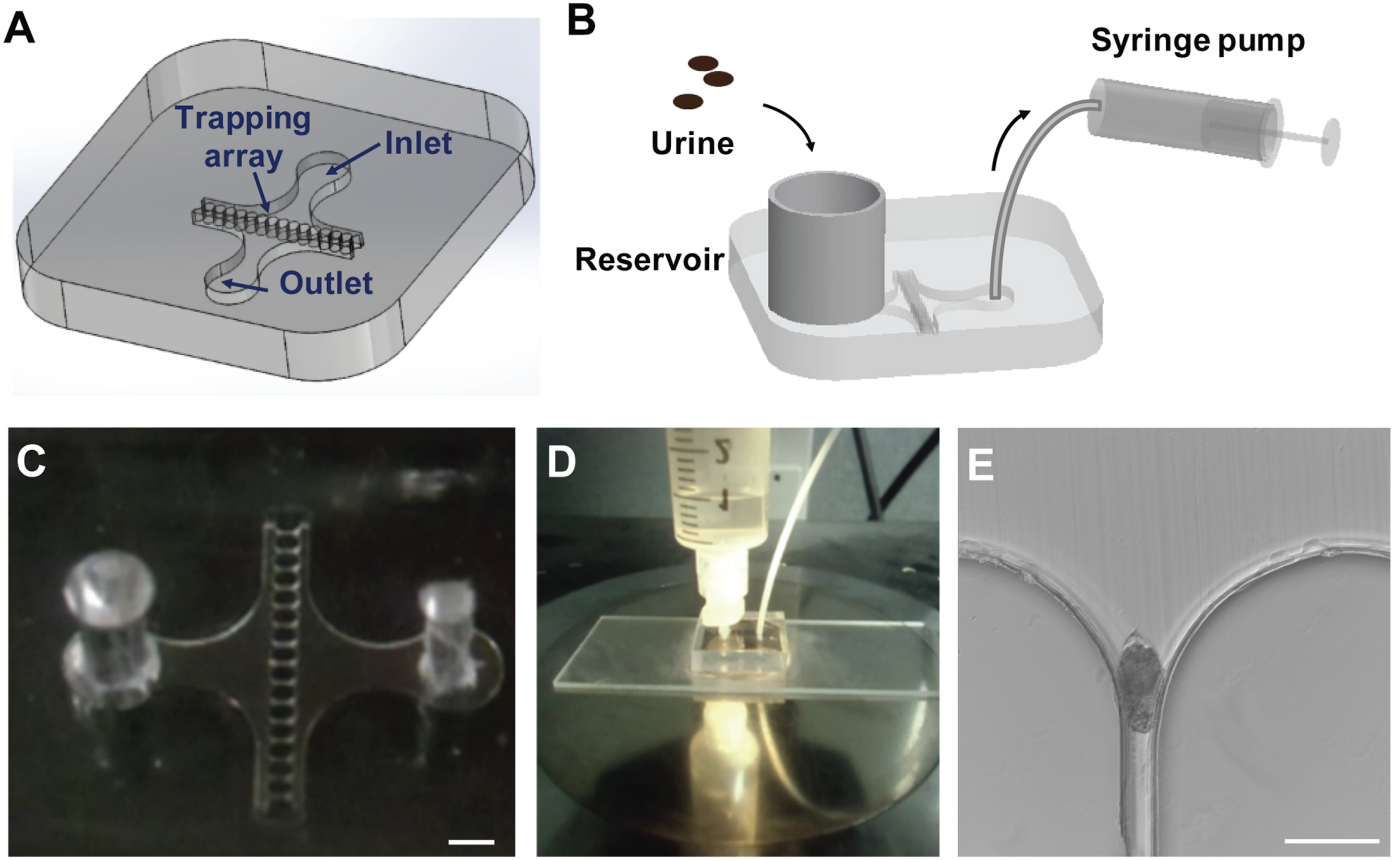
A microfiltration device for trapping and analysis of Schistosoma haematobium eggs. (A) Schematic of the microfiltration device. (B) Schematic representation of the experimental setup with a syringe pump withdrawing fluid from the outlet of the microchip. (C) Image of a fabricated microfiltration device. Scale bar, 1 mm. (D) Image of the microchip with a reservoir assembled. (E) Brightfield image of a schistosome egg captured in the microfluidic trap. Scale bar, 100 μm.

In this study, we design a microfiltration device to concentrate, isolate, and characterize schistosome eggs in urine (Fig 1). This system consists of a linear array of microfluidic traps in a PDMS channel. Computational fluid dynamics (CFD) is performed to study the uniformity of fluid flow and pressure drop in the microfluidic trap array for optimizing the throughput and trapping efficiency. The effects of flow resistance on sequential loading of schistosome eggs are evaluated for egg enumeration. The trapping efficiency is characterized under different operating conditions, including pumping direction and flow rate, to optimize the performance of the device. The applicability of the device for diagnosis of urogenital schistosomiasis is studied by isolating eggs of *Schistosoma haematobium* in urine. On-chip staining is also performed for in situ fluorescence analysis of the trapped eggs.

## Materials and Methods

### Preparation of schistosome eggs

Eggs of *Schistosoma haematobium* were isolated from experimentally infected hamsters (obtained from the Schistosome Research Reagent Resource Center and delivered by BEI Resources, NIAID, NIH) and suspended in normal saline (0.9% NaCl). The Institutional Animal Care and Use Committee approved all animal work and protocols.

### Microfluidic design and simulations

The microfiltration device was designed using Solidworks 2014 (Dassault Systemes SolidWorks Corp., MA). The device was constituted by a chamber with a linear array of 15 trumpet-shaped microfluidic traps, which immobilized eggs of different sizes at the opening. The dimension of the microfluidic trap was 100 μm in height and 25 μm in width in the narrow region. To evaluate the performance of the device, COMSOL Multiphysics (Comsol AB, Stockholm, Sweden) simulation was performed. The 3D Solidworks model was imported into COMSOL to generate the mesh. The steady-state Navier-Stokes equation was solved to analyze the flow field. The flow was laminar (Reynold’s number less than 1) and the boundary conditions consisted of a constant flow rate at the outlet and atmospheric pressure at the inlet. The no-slip boundary condition was assumed for other surfaces.

### Microfabrication of microfiltration devices

The microfiltration devices were fabricated by PDMS molding. The master mold was created by computer numerical control (CNC) machining with aluminum, which is rapid and cost-effective. The 3D Solidworks model was applied for CNC machining. The PDMS was prepared with a 10:1 elastomer base-curing agent ratio (w/w). The PDMS was degassed and poured onto the master mold to approximately 5 mm thick. The sample was cured at 65°C overnight. The PDMS channel layer was punched to create inlets and outlets, activated with air plasma and bonded to a glass microscope slide to seal the channel.

### Isolation of schistosome eggs

The outlet of the chip was connected to a syringe pump and a reservoir was attached to the inlet. A syringe pump was used to withdraw the liquid from the outlet. The microchip was prefilled with water before the experiment to minimize bubble formation. The device was loaded on the microscope stage and the sample was applied in the inlet reservoir. To minimize attachment of eggs on the channel surface, 0.1% Pluronics F127 (Sigma-Aldrich, St Louis, MO) was added to the sample. The trapping efficiency was calculated as: captured eggs / (captured eggs + bypassed eggs).

### Time-lapse microscopy

Time-lapse microscopy was performed to monitor the trapping process. Brightfield images were acquired at 1 sec intervals using a 2× objective lens on a Nikon Ti microscope. The microscope was equipped with HQ CCD camera (Photometrics, Tucson, AZ) and micromanager software (Vale Lab, University of California San Francisco).

### On-chip fluorescence labelling

The eggs were trapped in the microfiltration device hydrodynamically with a continuous flow of 1 μl/min. Fixation buffer (4% paraformaldehyde in normal saline) was introduced into the device and incubated with the eggs for 1 hour. Followed by washing with normal saline, the sample was incubated with Alexa Fluor 568 phalloidin and Sytox Green (Thermo Scientific, San Diego, CA) in 0.1% Triton-X100 in normal saline for 2 hours. The sample was washed before imaging.

### Confocal imaging

The stained eggs were examined using a Leica SP8 confocal microscope equipped with a 25× objective lens. Stacks of images for 3D rendering were taken at z-scaling of 0.6 μm. The confocal images were analyzed using the Imaris software (Bitplane, MN).

## Results and Discussion

### Design of the microfiltration device for trapping schistosome eggs

**Fig 2:**
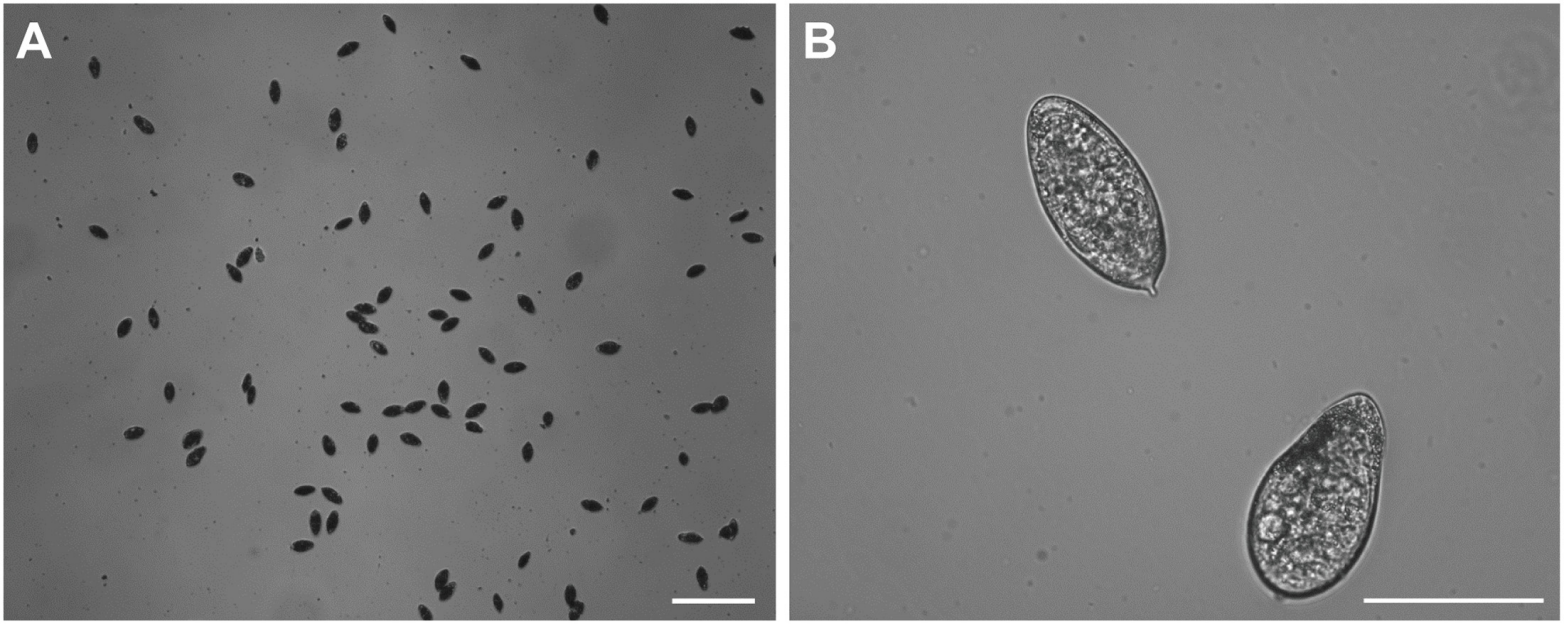
Bright-field images of *Schistosoma haematobium* eggs. Scale bars, 500 μm (A) and 100 μm (B).

The general design of the microfiltration device consists of a linear array of microfluidic traps in the chamber (Fig 1A). The geometry of the microfluidic trap is designed based on the size of eggs of *Schistosoma haematobium*. Fig 2. shows brightfield images of schistosome eggs, revealing the average and distribution of the size. The narrow region of the microfluidic trap is designed to be 25 μm, which is small enough to capture the eggs but large enough for other particles such as cell debris to pass through (S1 Fig).

The sample is applied to the inlet reservoir and a syringe pump is connected to the outlet for withdrawing the fluid or reversing the flow to retrieve the eggs (Fig 1B–1D). Eggs in the sample, such as urine, are captured in the trumpet-shaped microfluidic trap arrays (Fig 1E).

### Performance of the microfiltration device

**Fig 3:**
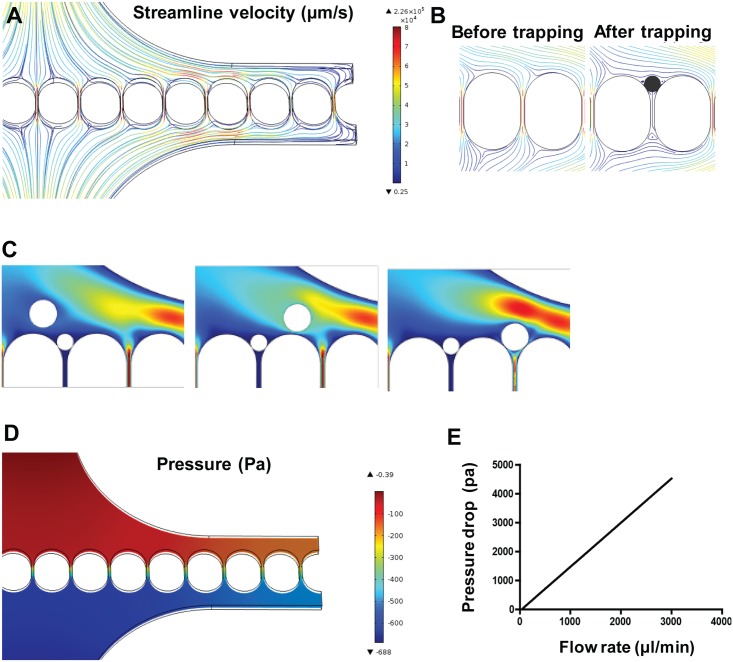
Computational fluid dynamics simulation. (A) Streamline velocity of the fluid flow in the microfiltration device. Color bar represents the fluid velocity. (B) Two-dimensional profiles of streamline velocity before and after trapping an egg. (C) 2D simulation of the sequential loading. After capture of the first egg, the following egg is diverted to the adjacent channel. The color represents the magnitude of velocity. (D) The pressure field in the cross-section in depth 50 μm of the chamber. Color bar represents the pressure. (E) Pressure drop across a trapping channel as a function of the volumetric flow rate.

To study the performance of the microfiltration device, we developed a 3D computational model of the microchannel (Fig 3A–3C). Under this model, the streamline velocity and pressure fields in the microchannel were studied. Without an egg, a relatively uniform flow field was observed among the linear array of microfluidic traps, thus the uniformity of the flow can evenly dispense the eggs into individual channels. Experimental characterization also confirmed uniform distribution of eggs in the microfluidic traps.

The pressure drop across the microfluidics traps generally increased with flow rate, which could increase the chance for the eggs to slip through. Fig 3D shows the pressure distribution with a flow rate of 300 μl/min. By increasing the flow rate to 3000 μl/min, the pressure drop across the channel could be over 4000 Pa (Fig 3E).

Experimentally, the eggs can be captured robustly in the trap in a flow rate of 300 μl/min without slipping through (S1 Video). Nevertheless, a large pressure drop with a high flow rate (*e*.*g*., 3000 μl/min) could push some eggs through the channel (S2 Video).

### Sequential loading of eggs by flow resistance selection

**Fig 4:**
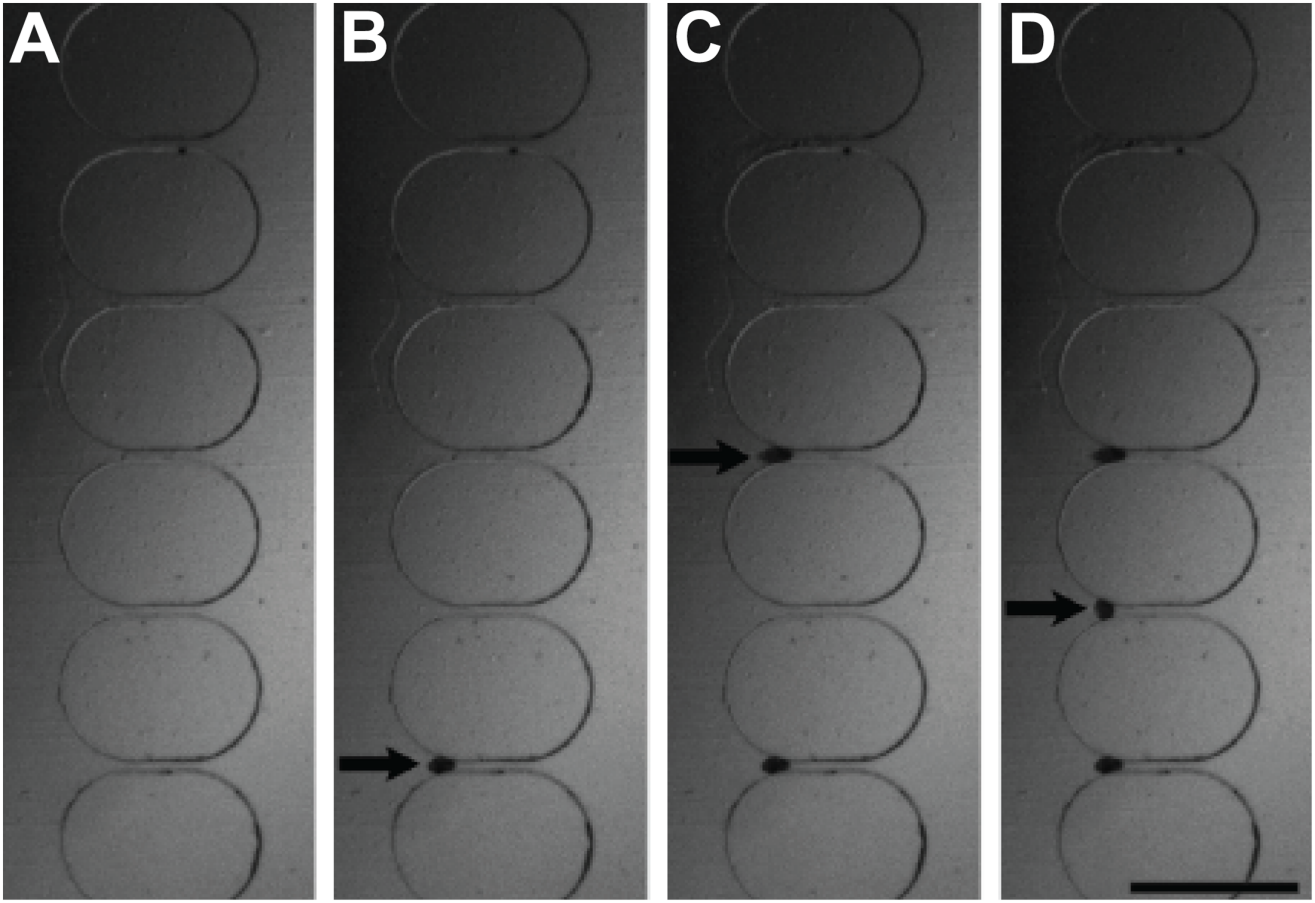
Sequential loading of eggs into the microfluidic traps. (A-D) Time-lapse images of *Schistosoma haematobium* eggs trapping. The fluid was flowing from left to right. Black arrows indicate trapping of schistosome eggs. The duration of the experiment was 60 sec. Scale bar, 500 μm.

The sequential loading of eggs is a useful feature of the microfiltration. The presence of the egg increased the flow resistance in the trapping port, which diverted the flow to other channels, enabling sequential loading of eggs. We investigated the sequential loading using CFD simulations. Under typical operating conditions (*e*.*g*., volumetric flow rate of 300 μl/min), the Reynold’s number was small (Re = 0.41) in our experimental condition, indicating laminar flow in the channel. Fig 3B shows the velocity field in the device with an egg trapped in a microfluidic trap. This design promoted sequential loading along the trapping array (Fig 3C). Fig 4 shows a representative trapping experiment with eggs sequentially loaded into the microfluidic traps (see also S3 and S4 Videos). The sequential loading facilitates optical analysis and counting of eggs in low abundance samples. For samples with a high concentration of eggs, multiple eggs could be captured at the trumpet-shaped microfluidic traps without blocking the channel (S2 Fig).

### Microfiltration of schistosome eggs

**Fig 5:**
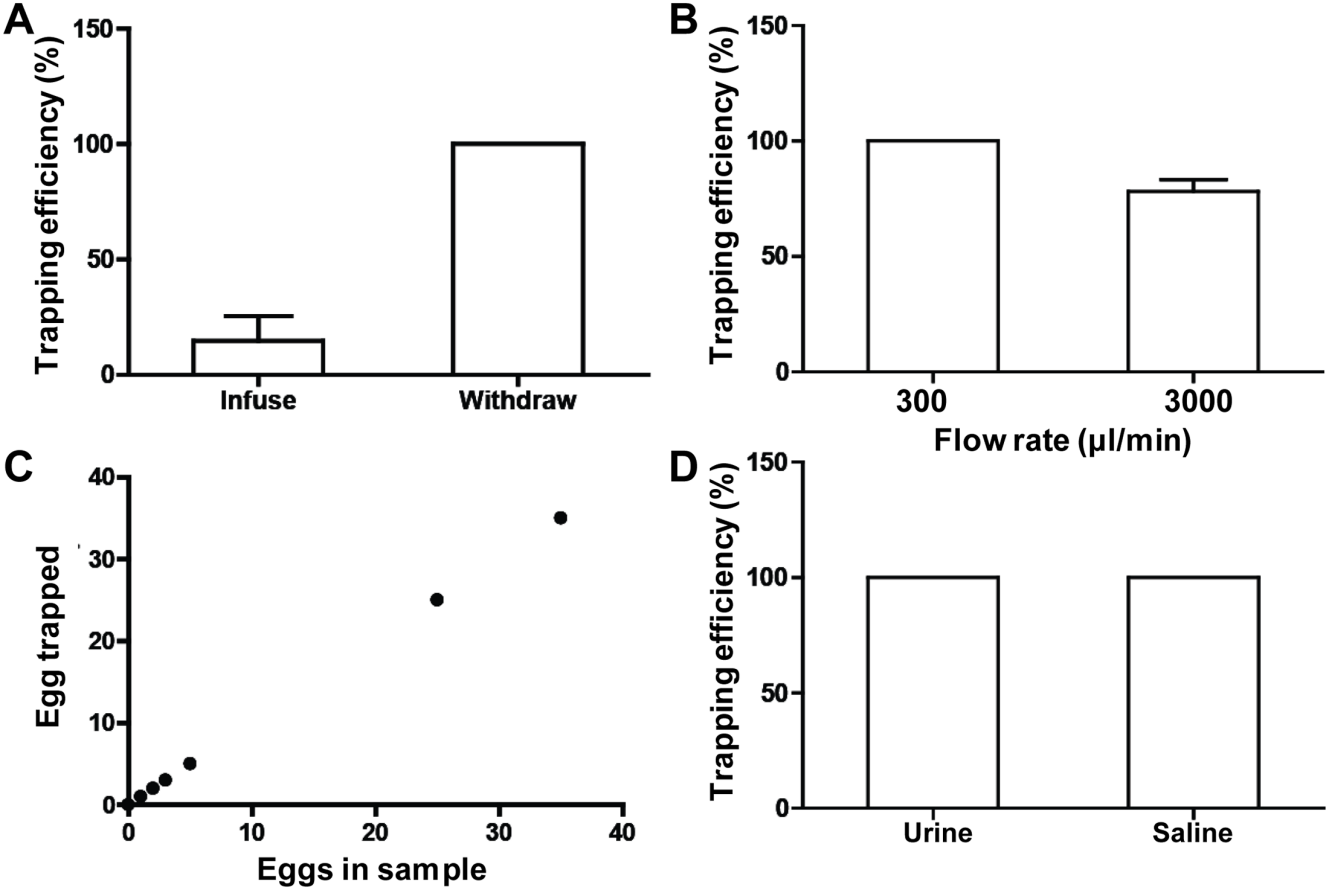
Schistosome eggs capture and enumeration. (A) Trapping efficiency of the microfiltration device by withdrawing samples from the outlet or infusing samples through inlet. (B) Trapping efficiency of the device at different flow rates. (C) Calibration of the trapping efficiency by spiking various number of egg into the sample. (D) Capture of schistosome eggs in urine and normal saline.

We further optimized the design of the system to increase the overall efficiency. A major consideration is egg sedimentation. Due to the size and density of schistosome eggs, when the sample was pushed into the inlet, a large number of eggs remained in the syringe instead of entering into the microfiltration device, resulting in a low trapping efficiency. To minimize the effects of sedimentation, the fluidic connection was shortened by creating a reservoir directly on top of the inlet (Fig 1B and 1C). The eggs were loaded into the device by withdrawing the fluid with a syringe pump in the outlet. The length of the syringe increased the sedimentation length, which minimized the retention and nonspecific adhesion of eggs to the syringe or tubing. Since the reservoir was directly attached on the device, the priming of the device could generate fluid motion in the sample reservoir, reducing the sedimentation loss. Comparing to infusing sample through the inlet, the trapping efficiency and recovery rate were improved dramatically using the reservoir and fluid withdrawal method (Fig 5A).

We further assessed the effects of flow rate on trapping efficiency. We applied a flow rate from 300 μl/min to 3000 μl/min, which required 1 to 10 minutes for processing the sample (Fig 5B). At a flow rate of 300 μl/min, the overall recovery is 100%. Due to the size and rigidity of schistosome eggs, they were unlikely to deform and slip through the trapping channels. However, a high pressure drop under high flow rate could drive the eggs to slip through the trapping spaces. At 3000 μl/min, the trapping efficiency reduced to approximately 83% due to slipping through of eggs across the microfluidic traps (S2 Video).

Depending on the severity of the infection, there could be less than 5 eggs per millilitre of urine [14]. In our experiment, 3 mL of samples at a concentration ranging from 0–12 eggs/mL was applied to evaluate the trapping efficiency of the device. Under 300 μl/min, the number of trapped eggs was consistent with different numbers of eggs spiked into the sample (Fig 5C). We also spiked eggs into urine samples derived from a healthy volunteer to demonstrate the feasibility of the device for urogenital schistosomiasis diagnostics. Comparing the trapping efficiency between normal saline buffer and urine samples (Fig 5D), our data indicated that the trapping efficiency was not affected by the sample type, supporting the feasibility of the device for urogenital schistosomiasis diagnostics.

**Fig 6:**
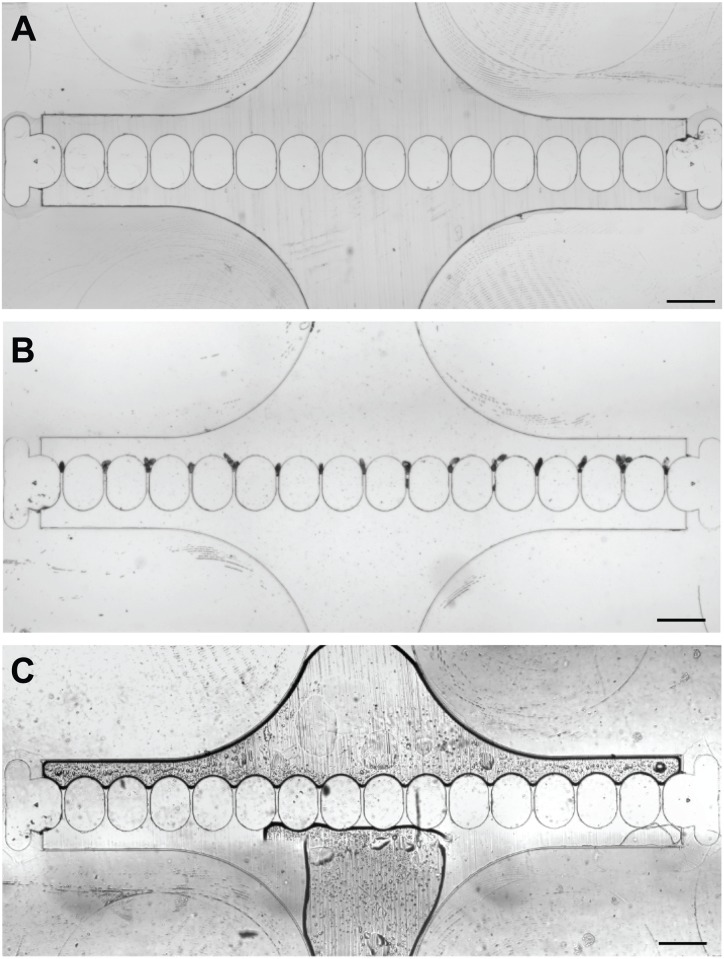
Recovery of schistosome eggs from the microfiltration device. (A) Micrographs of the microfluidic chamber before egg capture. (B) Schistosome eggs were captured in the microfluidic trap. (C) Recovery of schistosome eggs by pipetting the sample out from the inlet. Scale bars, 500 μm.

After filtration, the eggs could be recovered by directly pipetting through the inlet (Fig 6). Considering the volume of the microfluidic chamber (1.6 μl), the effective volume reduction achieved by the device was over 3 orders of magnitude. The eggs can then be utilized for subsequent molecular analysis such as PCR, culturing or immunoassay.

### On-chip staining and fluorescence analysis of schistosome eggs

**Fig 7:**
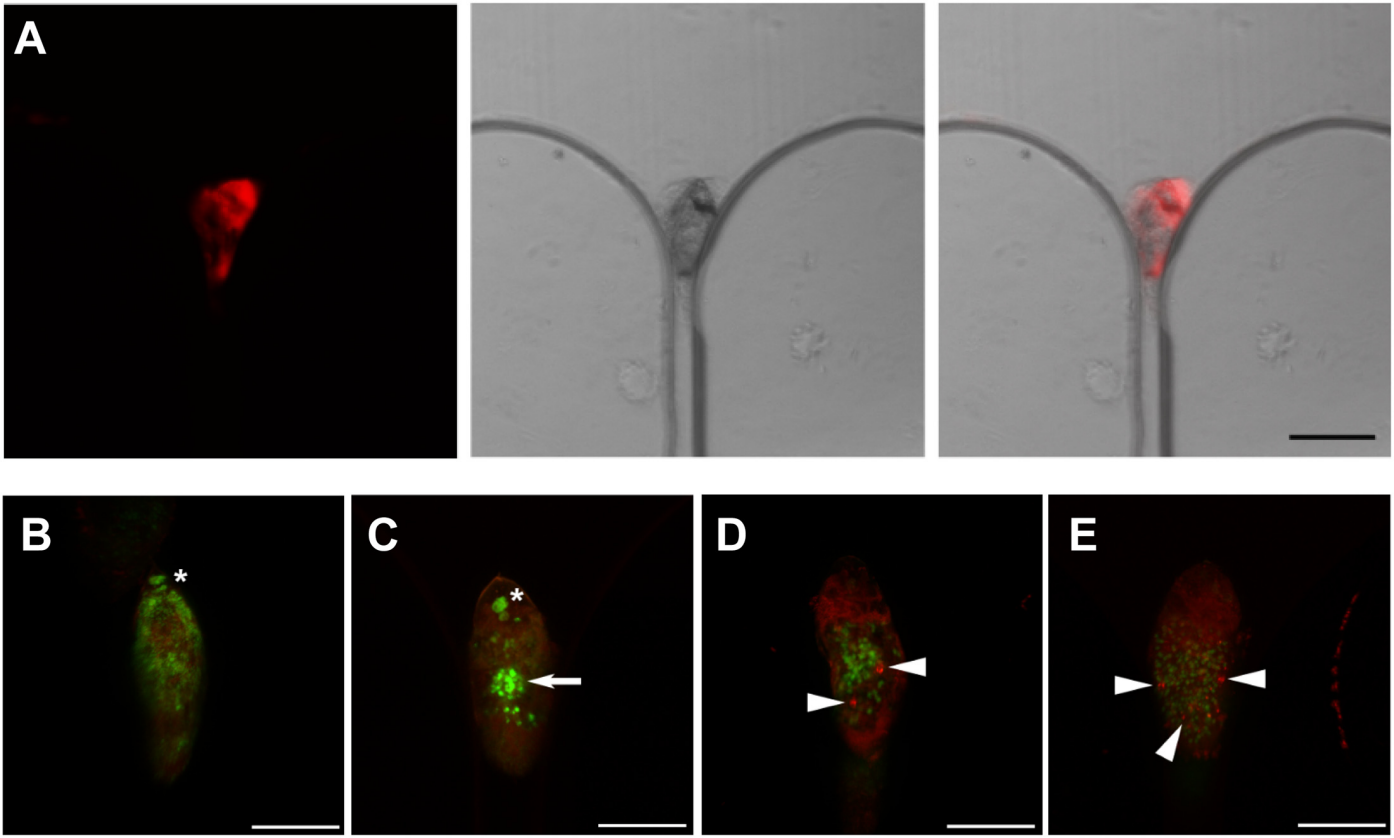
On-chip fluorescence characterization of schistosome eggs. (A) Fluorescence, brightfield and combined images of an egg capture in the microfluidic array. Scale bar: 100 μm. (B-D) Confocal fluorescence characterization of eggs of Schistosoma haematobium. Arrow and stars indicate the neural mass primordium and blastomere pairs. Arrow heads represent the flame cells. The eggs were double-labelled with phalloidin (red) and Sytox Green (green). Scale bars, 50 μm.

Compared with traditional membrane filtration methods, the microchip is compatible with microscopic characterization and provides a versatile means of acquiring the morphological information of the eggs. Individual eggs could be maintained in the microfluidic traps under a small flow (*e*.*g*., 1 μl/min), which facilitates in situ staining and on-chip analysis. The eggs of *Schistosoma haematobium* are characterized by the oval-shaped body (140 μm × 60 μm) with an external terminal spine aligning to the long axis of the egg (Fig 2B). Fluorescence characterization was performed by consecutively loading the fixation solution (4% paraformaldehyde) and washing solution (normal saline) into the chamber, followed by the staining solution consisting of phalloidin for F-actin staining and Sytox Green to label nuclei. Fig 7A. shows brightfield and fluorescence images of a trapped egg stained on-chip. The captured eggs in different stages of development could be identified by confocal imaging of nuclei and actin structures (Fig 7B–7E). In the confocal images, nuclei staining showed the neural mass primordium, which arises during early stages of organogenesis. We also observed flame-cell differentiation in some eggs which are in late stages of organogenesis. The blastomere pairs were identified by large round nuclei (Fig 7B–7E). Compared to conventional histological techniques [15], this device greatly simplified the staining procedure by enabling on-chip and in situ fluorescence characterization. The background noise was also reduced by confocal imaging across microchannels [16].

## Conclusions

In summary, we demonstrated a microfiltration device for schistosomiasis diagnostic applications. The system integrated trapping, isolation, enumeration, and imaging functions into a single chip. By incorporating with a cell phone or other low-cost imaging system, the device may potentially be applied in resource limited settings for point-of-care diagnostics [17, 18]. The device can also be combined with molecular biosensors for detection of urogenital schistosomiasis in the future [9, 19–22].

## Supporting Information

S1 Fig Brightfield images of eggs captured at the trumpet-shaped microfluidic traps. Scale bar, 100 μm.
(TIF)

S2 Fig A large number of eggs could be captured at the trumpet-shaped microfluidic traps. Scale bar, 500 μm.
(TIF)

S1 Video Capture of schistosome eggs by the microfiltration device. The flow rate was 300 μl/min. Brightfield images were acquired at 1 sec intervals. The duration of the video was 200 sec. Scale bar, 500 μm.
(MOV)

S2 Video Schistosome eggs slipping through the microfluidic traps under high pressure drop. The flow rate was 3000 μl/min. Brightfield images were acquired at 1 sec intervals. The video has a duration of 15 sec. Scale bar, 500 μm.
(MOV)

S3 Video 2D simulation of cell trapping. The color represents the magnitude of velocity.
(MOV)

S4 Video 2D simulation of sequential loading. After trapping of an egg, the existence of the egg leads to an increase in flow resistance and sequential loading of the second eggs. The color represents the magnitude of velocity.
(MOV)
