# Supplementary figures and images for: A Microfiltration Device for Urogenital Schistosomiasis Diagnostics
Source: PLoS One. 2016 Apr 28;11(4):e0154640. doi: 10.1371/journal.pone.0154640 (PMC4849660; doi:10.1371/journal.pone.0154640)

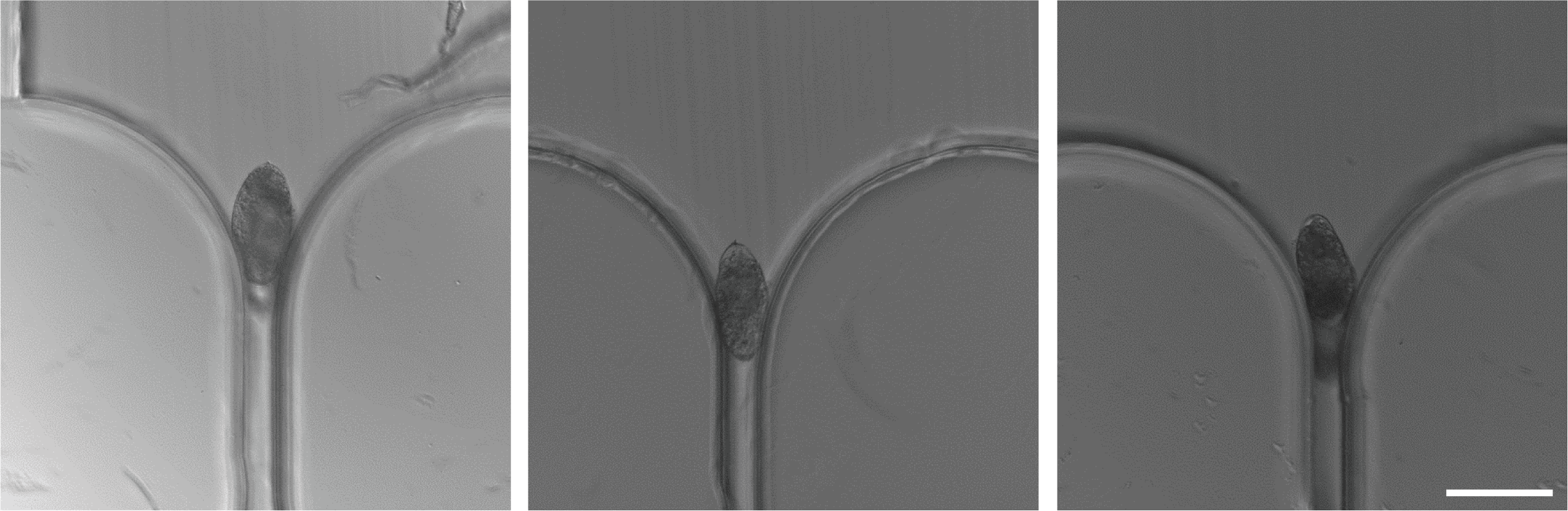

Supplement: S1 Fig — Scale bar, 100 μm. (TIF) [file pone.0154640.s001.tif]

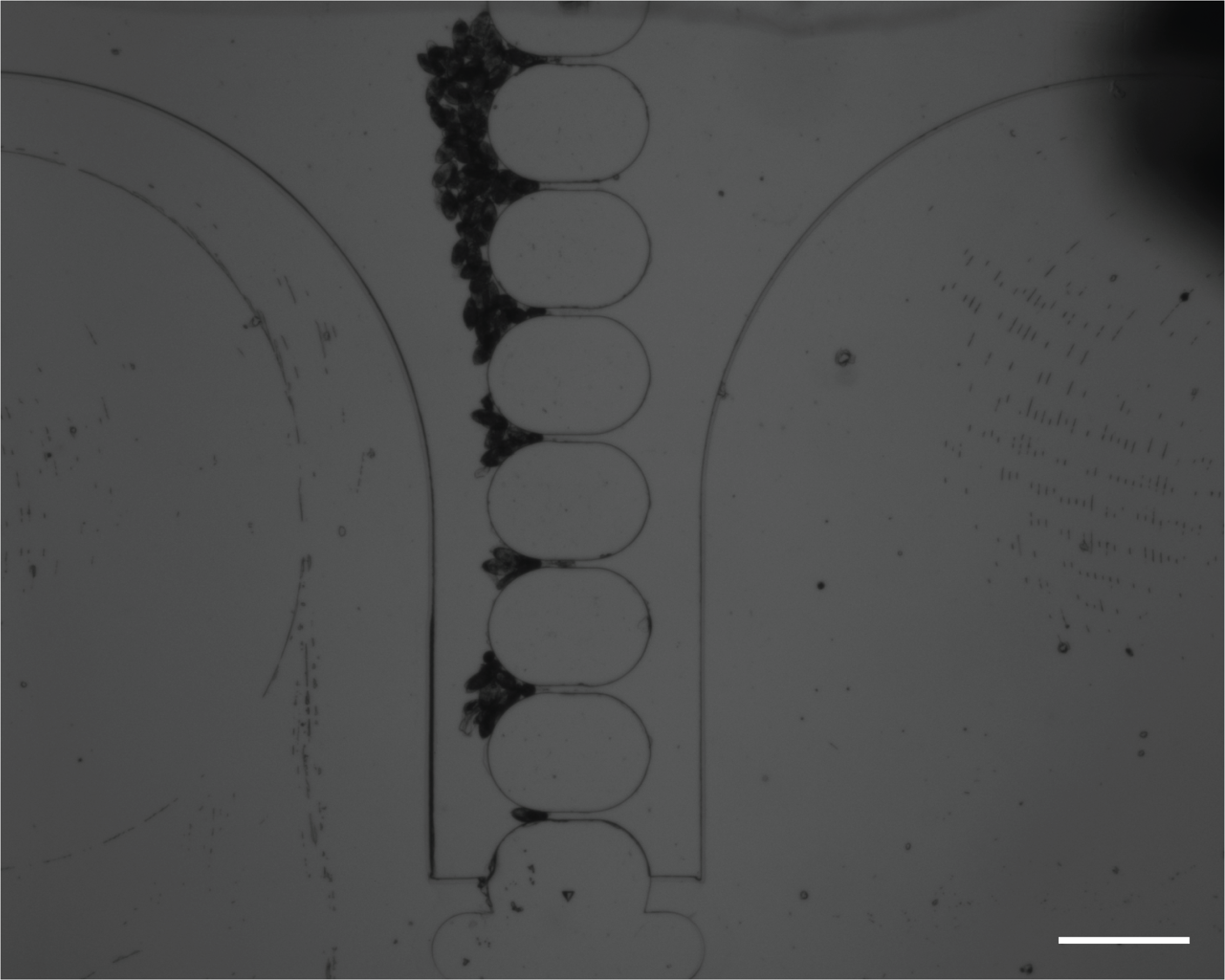

Supplement: S2 Fig — Scale bar, 500 μm. (TIF) [file pone.0154640.s002.tif]
